# 深共晶溶剂协同羧甲基-*β*-环糊精促进美托洛尔的毛细管电泳手性分离

**DOI:** 10.3724/SP.J.1123.2024.01024

**Published:** 2024-04-08

**Authors:** Xiaoqian LI, Zhining XIA

**Affiliations:** 重庆大学药学院, 重庆 401331; School of Pharmacy, Chongqing University, Chongqing 401331, China

**Keywords:** 毛细管电泳, 协同作用, 手性分离, 深共晶溶剂, 美托洛尔, capillary electrophoresis (CE), synergistic interaction, chiral separation, deep eutectic solvents (DESs), metoprolol (MET)

## Abstract

手性药物的对映体理化性质极其相似,而药理、毒理作用常常存在显著差异,因此探索提高对映体分离性能的创新策略对手性分离具有重要意义。以往关于毛细管电泳(CE)中*β*-受体阻断药美托洛尔(MET)对映体分离的研究主要集中在仅添加手性选择剂环糊精(CD)及其衍生物,而对于在CD的基础上添加辅助添加剂的研究较少。本研究将3种深共晶溶剂(DESs)氯化胆碱-葡萄糖、氯化胆碱-果糖和乳酸-葡萄糖作为辅助添加剂,用于MET的CE手性分离,推测了DES对基于CD的毛细管电泳分离MET对映体的协同作用。首先考察了CD种类、CD浓度、缓冲液pH值和缓冲液浓度对MET分离的影响,优化得到了最优条件(15 mmol/L羧甲基-*β*-环糊精、pH=3.0、40 mmol/L磷酸盐缓冲液)。其次制备了3种DESs作为添加剂,研究不同DESs及其质量分数对手性分离的作用,最终确定了最佳条件:采用质量分数1.5%的氯化胆碱-果糖型DES,此时MET的分离度从不添加DES时的1.30提升至2.61,实现了基线分离。最后对分离效果以及机理进行了推测。本文所建立的MET对映体手性分离分析方法不仅对提高手性药物质量、保证临床用药安全有效具有重要意义,还有助于将DES协同用于CD衍生物毛细管电泳手性分离的进一步研究与发展。

## Abstract (en)

The physical and chemical properties of chiral drugs are very similar. However, their pharmacological and toxicological effects vary significantly. For example, one enantiomer may have favorable properties whereas the other may be ineffective or even have toxic side effects. Hence, exploring innovative strategies to improve enantiomeric resolution is of great importance. Metoprolol (MET) is a *β*-receptor blocker used to treat hypertension, stable angina pectoris, and supraventricular tachyarrhythmia. Establishing chiral separation and analysis methods of MET enantiomers is important for enhancing the quality of chiral drugs. Capillary electrophoresis (CE) has the advantages of a small sample size, simple operation, high separation efficiency, and many alternative modes; therefore it is widely used in the field of chiral drug separation. The chiral selectors commonly used for CE-based chiral separation include cyclodextrin (CD) and its derivatives, polysaccharides, proteins, and macrocyclic antibiotics. CD is one of the most commonly used and effective chiral selectors for CE. The relatively hydrophobic structure inside the cavity and the relatively hydrophilic structure outside the cavity of CD enable it and chiral molecules to form inclusion compounds with different binding constants, thus achieving chiral separation. However, the use of CD alone as a chiral selector does not always yield satisfactory separation results. Hence, the addition of other additives, such as ionic liquids and deep eutectic solvents (DESs) to assist CD-based chiral separation systems has received extensive attention. Previous studies on the enantiomeric separation of MET by CE have focused on the addition of CD and its derivatives alone for separation. Few studies have been conducted on the synergistic addition of auxiliary additives to CD to improve the enantiomeric resolution of MET. In this study, three DESs, namely, choline chloride-D-glucose, choline chloride-D-fructose, and lactate-D-glucose, were used for the CE-based chiral separation of MET for the first time, and the synergistic effect of the DESs on the separation of MET enantiomers by CD-based capillary zone electrophoresis was speculated. For this purpose, an uncoated fused silica capillary with inner diameter of 50 μm, total length of 50 cm and effective length of 41.5 cm was used as the separation column. First, the effects of CD type, CD concentration, buffer pH, and buffer concentration on MET separation were investigated, and the optimal conditions (15 mmol/L carboxymethyl-*β*-cyclodextrin (CM-*β*-CD), pH=3.0, and 40 mmol/L phosphate buffer) were obtained. Other CE conditions were as follows: UV detection at 230 nm, applied voltage of 25 kV. All operations were carried out at 20 ℃. Next, three types of DESs were prepared as auxiliary additives via a mixed-heating method. The DESs were mixed in a 50 mL round-bottomed flask at a certain molar ratio and then heated in a water bath at 80 ℃ for 3 h until a clear and transparent liquid was obtained. The effects of different DESs and their mass fraction on chiral separation were subsequently studied. The optimal choline chloride-D-fructose mass fraction was ultimately determined to be 1.5%. The resolution of MET increased from 1.30 without DES to 2.61 with 1.5% choline chloride-D-fructose, thereby achieving baseline separation. Finally, the separation effect and mechanism were speculated. The MET chiral separation method established in this study is of great significance for improving the quality of chiral compounds and ensuring the safety and effectiveness of clinical drugs. Furthermore, it may be useful in the research and development of CE-based chiral separation techniques using CD derivatives with DESs.

对映异构体在自然界中普遍存在,其相关研究在医药领域尤为突出。虽然手性药物的对映异构体之间具有相同的化学结构,但它们在药理、毒理、药代动力学、代谢等生物活性方面存在明显差异,比如一个对映体有药效作用,而另一个无药效作用,甚至有毒副作用^[1,2]^。美托洛尔( MET,结构式见图1)是一种*β*受体阻断剂,临床上用于治疗高血压、稳定型心绞痛及室上性快速型心律失常。建立MET对映体的手性分离分析方法对提高手性药物质量、保证临床用药安全有效具有重要意义。

**图 1:**
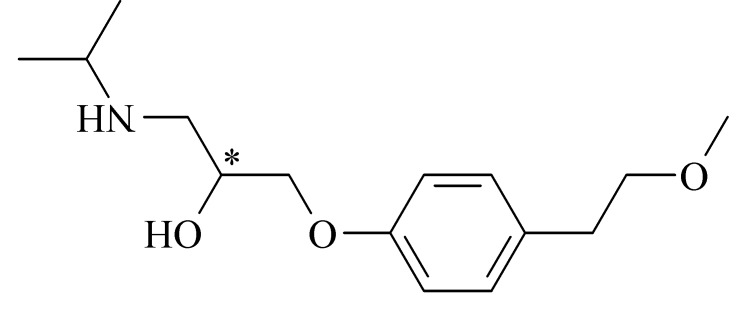
美托洛尔的结构式

目前,MET的手性分离方法主要是高效液相色谱法^[3,4]^,但其运行成本较高。毛细管电泳(capillary electrophoresis, CE)技术用于手性分离有进样量少、操作简单、分离效率高、可供选择模式多等优点,因此在手性药物分离领域应用广泛^[5⇓-7]^。毛细管电泳手性分离常选用的手性选择剂有环糊精(cyclodextrin, CD)及其衍生物、多糖、蛋白质和大环抗生素等。CD及其衍生物是毛细管电泳中最常用和最有效的手性选择剂之一,腔内相对疏水的结构和腔外相对亲水的结构使CD和手性分子形成不同结合常数的包合物,从而实现手性分离。然而,在某些情况下,单独使用CD作为手性选择剂并不总能提供令人满意的分离效果^[8,9]^。因此,在CD的基础上添加另一种添加剂(如离子液体、深共晶溶剂(DESs))协助手性分离的技术受到广泛关注^[10,11]^。DESs是由一定物质的量之比的氢键供体和氢键受体组成的低熔点混合物,又被称为低共熔溶剂^[12]^。目前,DESs主要应用于有机合成^[13,14]^、电化学^[15,16]^和分离萃取^[17⇓-19]^等方面。DESs的来源环保且成本低,基本不存在毒性,因此也有人将DESs称为“绿色溶剂”^[20⇓-22]^。

为了使MET对映体实现基线分离,本文拟运用毛细管电泳技术,考察CD种类、CD浓度、缓冲液pH值和缓冲液浓度对MET对映体分离的影响。同时制备3种DESs(氯化胆碱-葡萄糖(ChCl-D-glucose)、氯化胆碱-果糖(ChCl-D-fructose)和乳酸-葡萄糖(LA-D-glucose)),将其作为辅助添加剂,用于研究不同DESs及其质量分数对手性分离的作用。

本工作添加DESs协同羧甲基-*β*-环糊精(CM-*β*-CD)用于CE手性分离MET,分析DESs与CM-*β*-CD的协同作用,依据条件的变化,对效果以及机理进行了推测。本工作对其他将DESs协同作用于CD衍生物用于CE手性分离的工作具有借鉴意义。

## 1 实验部分

### 1.1 仪器与试剂

毛细管电泳仪(Agilent 7100 3D CE system,美国安捷伦科技有限公司);超声波清洗机(深圳市洁拓超声波清洗机厂家); pH计(Delta 320,瑞士梅特勒-托利多仪器公司);分析天平(AY 120,日本岛津公司);鼓风干燥箱(DGG-0670 A,上海齐欣科学仪器有限公司)。

磷酸二氢钠(NaH_2_PO_4_)、磷酸氢二钠(Na_2_HPO_4_)和磷酸(H_3_PO_4_)均为分析纯,购自上海阿拉丁生化科技股份有限公司;羟丙基-*β*-环糊精(HP-*β*-CD, ≥97%)、CM-*β*-CD(≥98%)、2,6-二-*O*-甲基-*β*-环糊精(DM-*β*-CD, ≥98%)均购自上海麦克林生化科技股份有限公司。氯化胆碱(ChCl, ≥99%)购自上海迈瑞尔化学技术有限公司;葡萄糖(DG)、果糖(DF)、乳酸(LA)均为分析纯,购自成都市科隆化学品有限公司;酒石酸美托洛尔消旋体(≥98%)由九鼎化学(上海)科技有限公司供应;去离子水购自屈臣氏集团(香港)有限公司。

### 1.2 DESs的制备

采用混合后加热的方法制备了3种不同的DESs。使用分析天平精密称取一定量的ChCl、DG、DF,使用移液枪吸取适量LA,分别按照ChCl(2.000 g)∶DG(2.839 g)=1∶1、ChCl(2.000 g)∶DF(2.581 g)=1∶1、LA(2 mL)∶DG(1.064 g)=5∶1的物质的量之比在50 mL圆底烧瓶中混合,于80 ℃水浴中加热3 h,直至得到均一透明的液体。冷却至室温后,将DESs封装于阴凉处保存。

### 1.3 溶液配制

用H_3_PO_4_调节pH配制一系列浓度和酸度的NaH_2_PO_4_-Na_2_HPO_4_缓冲液,分别向其中加入一定量的HP-*β*-CD、CM-*β*-CD、DM-*β*-CD和各类型DESs制备运行缓冲液,经0.25 μm微孔滤膜过滤后备用。

精密称取酒石酸美托洛尔标准品适量,用超纯水溶解并制成浓度为30 mmol/L的储备液,于4 ℃冰箱密封保存备用。精密量取储备液适量,用含各类CD衍生物和DESs的运行缓冲液稀释成3.0 mmol/L,经0.25 μm微孔滤膜过滤后,得到MET样品溶液。

### 1.4 CE的条件与方法

使用外径365 μm、内径50 μm、总长度50 cm、有效长度41.5 cm未涂覆的熔融石英毛细管,毛细管柱首次使用前用甲醇冲洗10 min, 1.0 mol/L NaOH冲洗2 h以进行活化。每次电泳运行之间,将毛细管依次用0.1 mol/L HCl、水、0.1 mol/L NaOH和运行缓冲液分别冲洗60 s以提高重复性。所有操作均在20 ℃下进行,进样方式选择压力进样5 s, 3000 Pa,运行电压均为25 kV,紫外检测波长为230 nm。优化后的缓冲液条件如下:15 mmol/L CM-*β*-CD, pH=3.0, 40 mmol/L磷酸盐缓冲液,1.5% ChCl-DF。

## 2 结果与讨论

### 2.1 CD种类对MET分离度的影响

运用毛细管电泳进行手性分离时需要借助手性选择剂,手性选择剂需要其与对映体之间能够发生相互作用,生成一定强度的包合物,且对映体形成的包合物会因相互作用强度的差异而存在淌度差。

选择10 mmol/L HP-*β*-CD、DM-*β*-CD和CM-*β*-CD分离MET对映体,从图2可以看出,HP-*β*-CD和DM-*β*-CD对MET没有拆分能力,仅CM-*β*-CD有拆分效果,推测是因为HP-*β*-CD和DM-*β*-CD呈电中性,而CM-*β*-CD带负电,MET在pH值为3.0的环境下带正电,二者发生静电相互作用,分离度提升,因此选择CM-*β*-CD为MET的手性选择剂。

**图 2:**
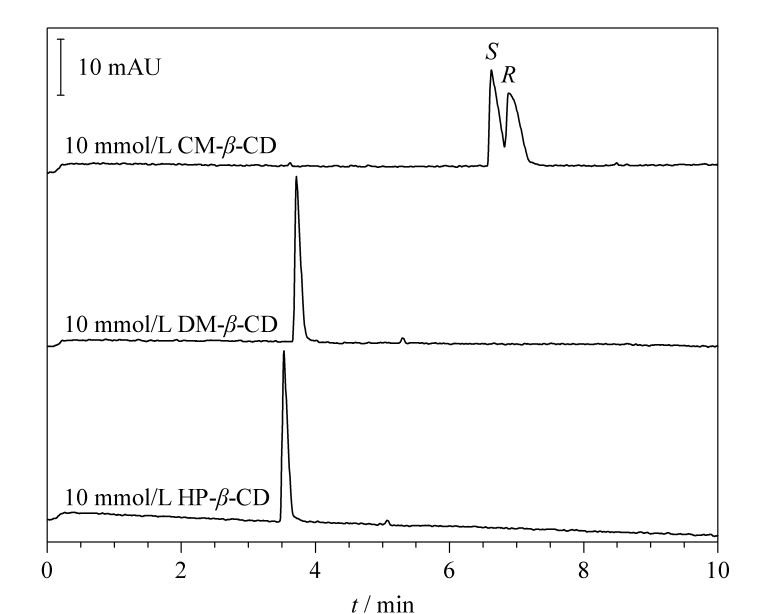
采用不同CD时美托洛尔的电泳图

另外,可以看到添加CM-*β*-CD有分离效果但并未达到基线分离,推测该手性选择剂与MET两个对映体之间相互作用产生的淌度差不足以实现基线分离。

### 2.2 CD浓度对MET分离度的影响

根据图3所示,随着CM-*β*-CD浓度的增加,手性选择剂与MET的浓度比增加,MET分离度增加,CM-*β*-CD浓度为15 mmol/L时分离度最大,浓度增加到20 mmol/L时,运行缓冲液黏度增加,使作用时间延长,导致峰展宽,柱效降低,分离效果变差。因此选择15 mmol/L为最佳CD浓度。

**图 3:**
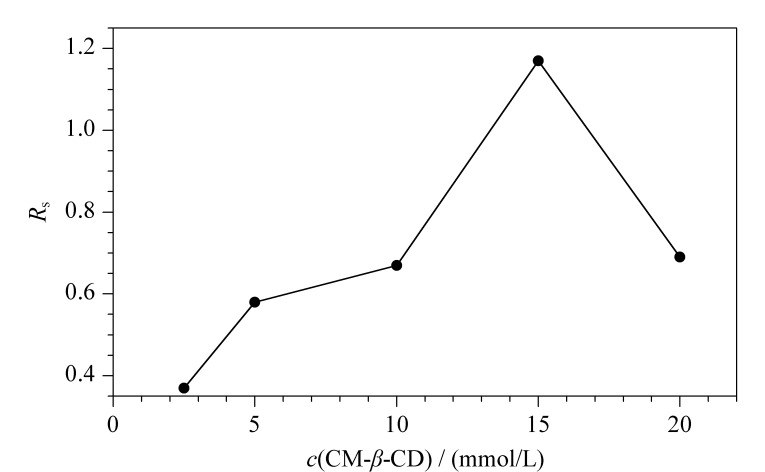
CM-*β*-CD浓度对美托洛尔分离度的影响

### 2.3 缓冲液pH值对MET分离度的影响

由图4可知,MET在pH=3.0时取得最佳分离度。由于电渗流的存在,pH=2.5时分离度小于pH=3.0时。因此MET的最佳分离pH值为3.0。

**图 4:**
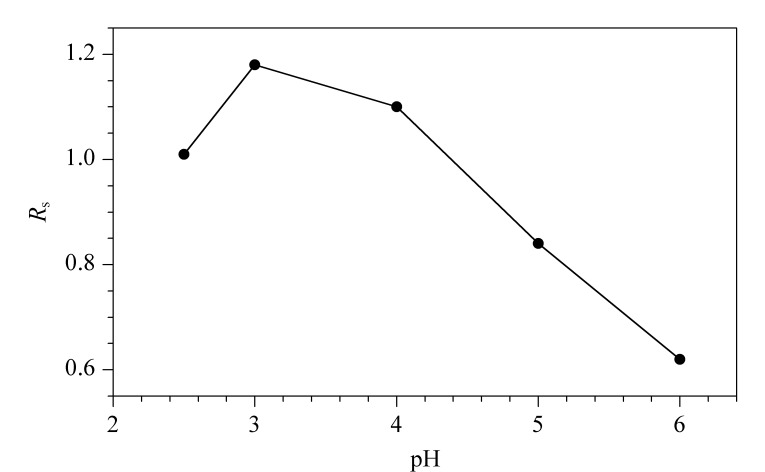
缓冲液pH值对美托洛尔分离度的影响

### 2.4 缓冲液浓度对MET分离度的影响

随着磷酸盐缓冲液浓度的增加,MET迁移时间逐渐延长,对映体的峰展宽明显。从图5可知,缓冲液浓度的增加会使MET的分离度增加,当浓度大于40 mmol/L时,电流也会随浓度增加,焦耳热增加。考虑到加入DESs作为辅助添加剂,它具有一定的离子强度,也会带来电流增加,故选择磷酸盐缓冲液的浓度为40 mmol/L。

**图 5:**
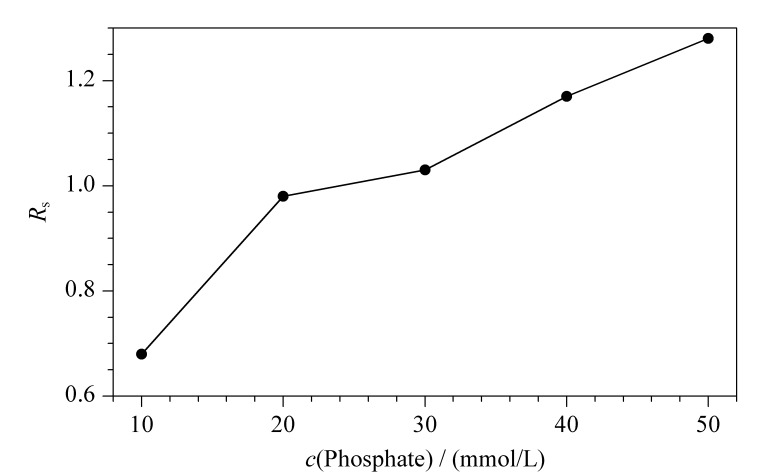
缓冲液浓度对美托洛尔分离度的影响

### 2.5 DESs种类对MET分离度的影响

DESs可以通过抑制CE电渗流、改变运行缓冲液的离子强度等作用与CD协同对MET进行手性分离。不同结构的DESs在理化性质上存在差异,比如极性、黏度等,这些差异可能对DESs与CD的协同作用产生影响。因此,在上述获得的最优条件下,分别加入占缓冲液1.0%质量分数的ChCl-DG、ChCl-DF、LA-DG 3种DESs作为辅助添加剂,考察DESs种类对CE手性分离的影响。

由图6可知,3种微量DESs均能明显改善分离度,使分离度均大于1.50,达到基线分离。加入3种DESs后迁移时间增加,推测其中一个原因是背景缓冲液黏度增加。而对映体分离度的增加主要是因为DESs的协同作用。综合考虑分析时间和分离度,ChCl-DF型DES分离效果最优。

**图 6:**
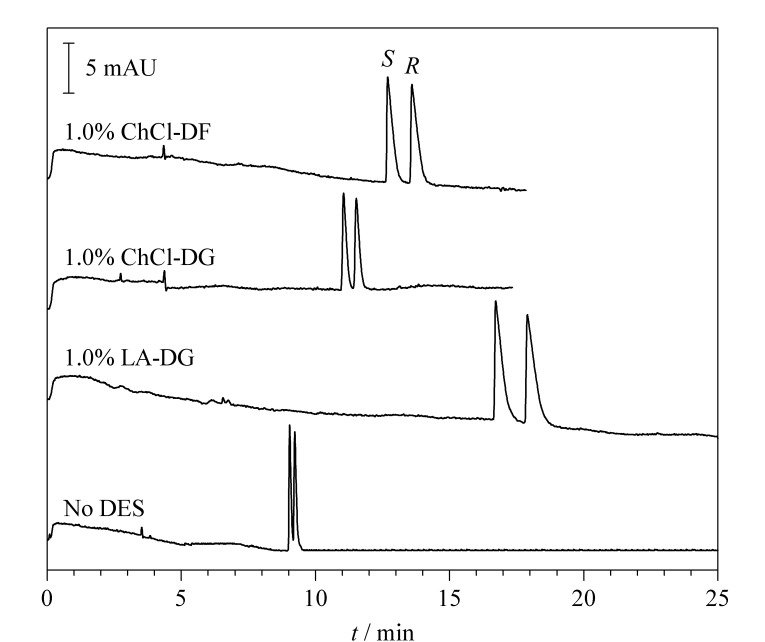
使用不同DESs时美托洛尔的电泳图

### 2.6 DESs质量分数对MET分离度的影响

考察了ChCl-DF质量分数对CE手性分离的影响。由图7可知,随着ChCl-DF质量分数的增加,MET分离效果逐渐增强,但同时电流也会随之增加,考虑到焦耳热会对分离产生不良影响甚至会使毛细管断裂,故不能进一步增加DES的质量分数。质量分数从1.0%增加到1.5%, MET分离度显著提升,再结合分析时间,选择1.5%作为最佳添加剂质量分数。此时,MET的分离度从不加DES时的1.30提高至2.61(见图7),实现了基线分离。

**图 7:**
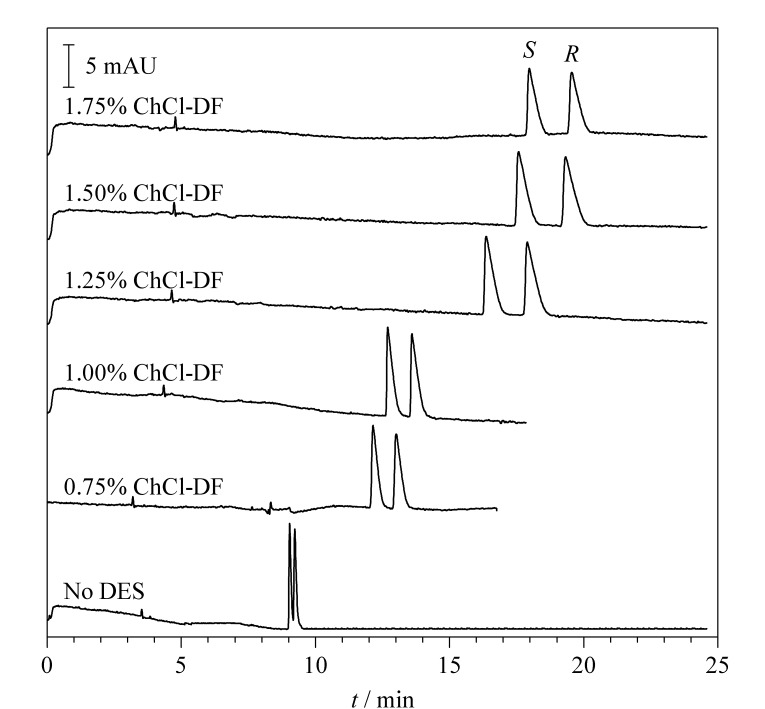
不同氯化胆碱-果糖质量分数下美托洛尔的电泳图

### 2.7 手性分离机理

据文献[23]报道,在DESs中加入具有宿主性能的物质可以提高其主客体包合作用。CD是一类由6~12个D-吡喃葡萄糖单元组合而成的环状低聚糖,其亲水性外壳和疏水性空腔可以通过非共价作用将几何尺寸合适的疏水性小分子包合在疏水空腔内形成主客体的包合物,温度、离子强度、极性等因素均可以对CD的包合作用产生影响。这种包合作用使得CD被广泛应用于医药、食品、化工和环境检测行业。

Moufawad等^[24]^通过紫外可见光谱法和静态顶空气相色谱法证明了在DESs中,CD仍然保留包合作用,有利于CD吸收挥发性有机化合物(VOC)。Di Pietro等^[25]^使用多重核磁共振技术证明了在纯DESs和DESs与水的混合物中*β*-CD可以与两种VOCs形成包合物。包合物诱导产生的化学位移变化和分子间主客体效应提供了在DESs中*β*-CD保留包合作用的证据。本文推测DES在CD与对映体之间产生挤压效应,对映体进入CD腔体的程度增加,即DES促进了CD对MET的相互作用,并且使两个对映体形成的CD包合物的淌度差增加,MET手性电泳的分离度得到提升。

## 3 结论

本文研究了DESs作为添加剂对毛细管电泳手性分离MET的影响。实验证明,DESs可以提高MET消旋体的分离效率。DESs能够提高毛细管电泳手性分离效率的可能原因如下:(1)DESs加入缓冲液后,离子强度会随之增加,使分离度得到提升。(2)DESs对CD和对映体具有挤压效应,可以提高CD的包合能力,对映体进入CD腔体的程度增加,从而提高分离度。今后,可结合计算化学的分子对接技术,进一步探索DESs作为添加剂改善毛细管电泳手性分离的机理。
